# A review on low-dimensional physics-based models of systemic arteries: application to estimation of central aortic pressure

**DOI:** 10.1186/s12938-019-0660-3

**Published:** 2019-04-02

**Authors:** Shuran Zhou, Lisheng Xu, Liling Hao, Hanguang Xiao, Yang Yao, Lin Qi, Yudong Yao

**Affiliations:** 1 Sino-Dutch Biomedical and Information Engineering School, Northeastern University, Shenyang, 110819 China; 2 Neusoft Research of Intelligent Healthcare Technology, Co. Ltd., Shenyang, 110167 China; 3 Chongqing Key Laboratory of Modern Photoelectric Detection Technology and Instrument, School of Optoelectronic Information, Chongqing University of Technology, Chongqing, 400054 China

**Keywords:** Physics-based model, Systemic arteries, Central aortic pressure, 0D model, 1D model, Tube-load model

## Abstract

The physiological processes and mechanisms of an arterial system are complex and subtle. Physics-based models have been proven to be a very useful tool to simulate actual physiological behavior of the arteries. The current physics-based models include high-dimensional models (2D and 3D models) and low-dimensional models (0D, 1D and tube-load models). High-dimensional models can describe the local hemodynamic information of arteries in detail. With regard to an exact model of the whole arterial system, a high-dimensional model is computationally impracticable since the complex geometry, viscosity or elastic properties and complex vectorial output need to be provided. For low-dimensional models, the structure, centerline and viscosity or elastic properties only need to be provided. Therefore, low-dimensional modeling with lower computational costs might be a more applicable approach to represent hemodynamic properties of the entire arterial system and these three types of low-dimensional models have been extensively used in the study of cardiovascular dynamics. In recent decades, application of physics-based models to estimate central aortic pressure has attracted increasing interest. However, to our best knowledge, there has been few review paper about reconstruction of central aortic pressure using these physics-based models. In this paper, three types of low-dimensional physical models (0D, 1D and tube-load models) of systemic arteries are reviewed, the application of three types of models on estimation of central aortic pressure is taken as an example to discuss their advantages and disadvantages, and the proper choice of models for specific researches and applications are advised.

## Introduction

Cardiovascular diseases have become a dominant factor of mortality all over the world [1]. Nearly 17.5 million people die of cardiovascular disease [2] and billions of dollars are spent every year on related healthcare [3]. Nowadays, cardiovascular research has become an important topic and been paid significant attention by researchers. Cardiovascular system is a complex circulatory system consisting of the heart, arteries and veins [4]. In recent years, due to the significant improvements in computer technology, modeling based on physical principles has become a powerful tool to simulate the hemodynamic properties of cardiovascular system and has been playing an increasingly important role in the diagnosis of cardiovascular diseases and the development of medical devices [5–7].

**Fig. 1:**
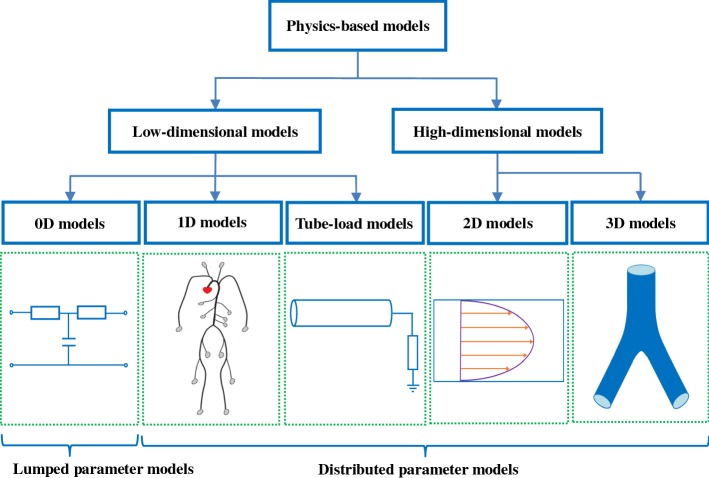
The structure diagram of physics-based models in the cardiovascular system

Current physics-based models can be divided into two categories, high-dimensional models and low-dimensional models as shown in Fig. 1. High-dimensional models including 2D models and 3D models can give detailed descriptions of the local flow field of the blood. These models describe the complex hemodynamic phenomenon of a specific region in the cardiovascular system. 2D models are generally used to describe changes of the radial blood flow velocity in an axisymmetric tube [8, 9]. 3D models are usually applied to simulate the fluid-structure interaction between the vascular walls and blood [10, 11]. To establish a 3D model of the entire arterial tree, the complex geometrical and mechanical information needs to be provided, which results in the enormous computational complexity, so that it cannot be readily implemented in practice. Consequently, high dimensional models can generally be used to simulate local hemodynamics of specific arterial sites, instead of the whole arterial tree.

In contrast to high-dimensional models, low-dimensional models with small computational costs can readily reproduce the pulse wave propagation phenomenon and realize patient-specific modeling. Thus, the low-dimensional modeling can be an effective way to describe the hemodynamic properties of the entire arterial tree in practical applications. At present, the available low-dimensional models mainly consist of 0D models, 1D models and tube-load models. 0D models, also called lumped parameter models, can describe global properties of the arterial system. The lumped parameter models are characterized by their pulse waveforms as a function of time only. The most well-known lumped parameter model is the Windkessel model [12], which includes mono-compartment models and multi-compartment models [13, 14]. 1D models and tube-load models are distributed parameter models, which can represent distributed properties of the arterial system. In the latter two types of models, their pulse waveforms depend on both time and space. In the distributed parameter models, 1D model based on the simplified Navier–Stokes equation is commonly used to reproduce pressure and flow at any position in the entire arterial tree [15–19]. The Windkessel model is computationally simple but less accurate. On the other hand, 1D model can represent the wave propagation phenomenon accurately but need a relatively large amount of computation. Taking advantage of both Windkessel models (simplicity) and 1D models (accuracy), some researchers developed tube-load models [20, 21]. Tube-load models can monitor multiple arterial hemodynamic parameters such as pulse transit time, arterial compliance, pulse wave velocity, and so on.

So far, these three types of low-dimensional models have been extensively used in the study of cardiovascular dynamics as shown in Table 1. Especially, applying physics-based models to estimate central aortic pressure has been paid much attention in recent decades [22–27]. However, to our best knowledge, there has been few review paper about the reconstruction of central aortic pressure using these physics-based models. Additionally, estimating central aortic pressure is a common application of these three types of models, which contributes to compare their advantages and disadvantages fully. Thus, this paper is to review three types of low-dimensional physics-based models (0D models, 1D models and tube-load models) of the arterial system and take the application of estimating central aortic pressure as an example to compare their advantages and disadvantages. To begin with, the theories and applications of Windkessel models including mono-compartment and multi-compartment models are described. Then, the theories and applications of two types of distributed parameter models, namely 1D models and tube-load models, are elaborated. Next, the advantages and disadvantages of these three models are discussed. Finally, future challenges and final conclusion are presented.

**Table 1:** Main applications of Windkessel models, 1D models and tube-load models

Model type	Main applications	References
Windkessel models	Estimation of cardiac output	[32, 94]
	Estimation of total peripheral resistance	[112]
	Estimation of total arterial compliance	[30, 33, 113]
	Estimation of aortic input impedance	[114, 115]
	Estimation of stroke volume	[116, 117]
	Estimation of central aortic pressure	[22, 23, 44–48]
	Providing outflow boundary condition	[34, 50, 60, 97]
	Physiological simulation of circulatory system	[118–120]
	Pathological simulation of circulatory system	[121–123]
1D models	Simulation of pulse wave propagation dynamics	[18, 34, 97, 124–127]
	Wave intensity analysis	[128–130]
	Estimation of central aortic pressure	[24, 25, 68, 69]
	Assessing the performance of algorithms and indexes	[131–133]
Tube-load models	Estimation of pulse wave transit time/velocity	[21, 81, 134, 135]
	Calculation of forward and backward waves	[77, 136, 137]
	Estimation of artery stenosis and stiffness	[134, 138]
	Prediction of the change of vessel diameter	[139]
	Estimation of central aortic pressure	[26, 27, 71–74, 90, 93]

## 0D models

In 0D models (lumped parameter models), the Windkessel theory is applied to the modeling of the arterial system [12, 28, 29]. Windkessel models are divided into two classes: mono-compartment models and multi-compartment models. Theories and applications of two categories of models are elaborated in this part, respectively. Furthermore, the comparison of different Windkessel models is made in Table 2.

**Table 2:** Comparison of different Windkessel models

Model type	Mono-compartment model			Multi-compartment model
	Two-element model	Three-element model	Four-element model	
Strengths	The model structure is simplest	The model can describe high frequency effects	The model can represent all the frequency effects well	The model can represent pulse wave propagation
Weaknesses	The model cannot represent high frequency effects	There is small error at low frequency	Parameter estimation is difficult	The model structure is more intricate
	The model cannot describe pulse wave propagation			

### Model descriptions

#### Mono-compartment models

The mono-compartment model is a combination of inductance, compliance and resistance. According to the number of elements included, current mono-compartment models are classified into four main types: two-element, three-element, four-element and complex mono-compartment Windkessel models.

##### a. Two-element Windkessel model

**Fig. 2:**
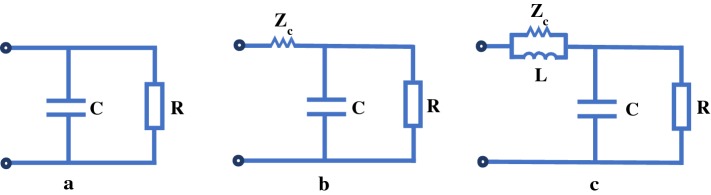
The mono-compartment models. **a** Two-element Windkessel model; **b** three-element Windkessel model; **c** four-element Windkessel model

The two-element Windkessel model is the simplest mono-compartment model presented by Frank [12], which is made up of a resistor (R) and a capacitor (C) as shown in Fig. 2a. In this model, the resistor describes the resistance of small peripheral vessels and the capacitor describes the distensibility of large arteries. The two-element Windkessel model simply describes the pressure decay of the aorta in diastole. This model cannot signify the high frequency effects because there is merely a time constant in the model. Owing to its simplicity, this model can be used in clinical practice readily such as total arterial compliance estimation [30] and blood pressure estimation [31].

##### b. Three-element Windkessel model

Adding a characteristic impedance ($Z_c$) to the two-element Windkessel model, the three-element Windkessel model is formed as shown in Fig. 2b [15]. The characteristic impedance is equal to oscillatory pressure divided by oscillatory flow. Although it is found that a resistance numerically equals approximately a characteristic impedance, the characteristic impedance is different from the resistance. The characteristic impedance is merely used to signify oscillatory phenomena. Owing to the inclusion of the characteristic impedance, this model can simulate high frequency effects. Simultaneously, the introduction of the characteristic impedance also results in some errors at the low frequency. In contrast with the two-element Windkessel model, the three-element Windkessel model can have a higher accuracy. Therefore, the three-element Windkessel model has been extensively used in theoretical research [32–34].

##### c. Four-element Windkessel model

Taking the inertance of blood flow into consideration on the basis of the three-element Windkessel model, Stergiopulos et al. [35] proposed a four-element Windkessel model as shown in Fig. 2c. Due to the addition of the inertance, this model can represent middle frequency effects. In other words, the four-element Windkessel model can simulate all frequency effects. It has been validated that the four-element model can give a better description of the impedance characteristics [36]. Some nonlinear regression analysis methods are applied to the estimation of the four-element model parameters. Compared with the two-element and three-element Windkessel models, it is more difficult to identify the model parameters of the four-element Windkessel model. Consequently, only a few researchers make use of this model [23, 37, 38].

##### d. Complex mono-compartment models

For the sake of the further improvement of the arterial model, a few researchers developed more complex Windkessel models in which more resistive, inductive and capacitive components were introduced [39, 40]. By including more resistive and inductive elements, the laminar oscillatory flow impedance can be simulated. Owing to the high complexity of these models, there has been no further development by other investigators.

These models described above focus on simulating the pressure and flow characteristics of the arterial vessels without considering the effect of the venous vessels. In fact, with regard to the coronary and pulmonary circulation, the pressure and flow of the veins have a significant impact on the global hemodynamics [41]. Under this circumstance, the venous side cannot be ignored. In order to describe the characteristics of the veins, extra resistance, inertance and compliance are added to form more complex (five, six and seven element) arterial models. In contrast with the two, three and four element Windkessel model, the five-element model simulates the characteristics of microcirculation hemodynamics more accurately, and the six-element model accounts for the hemodynamic contribution of the venous vessels in the cardiovascular system more precisely, and the seven-element model further gives the representation of the systemic circulation through the improvement on the description of the venous system.

#### Multi-compartment models

**Fig. 3:**
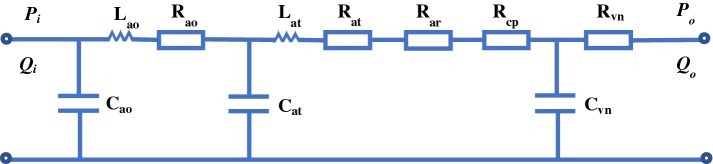
A multi-compartment model of systemic circulation. *ao* aortic root, *at* artery, *ar* arteriole, *cp* capillary, *vn* vein

Regardless of spatial information, the mono-compartment model regards all arteries as a single block. In order to represent the distribution of flow and pressure, some multi-compartment models which were composed of a series of mono-compartment models were established. Figure 3 is an example of a simple multi-compartment model of the systemic arteries [42]. Every mono-compartment model is a combination of resistance (R), inertance (L) and compliance (C). At present, there are four typical compartmental configurations in the multi-compartment model: T, $\Pi$ and inverted L element, respectively [13]. The corresponding compartmental configuration should be chosen appropriately according to the characteristics of the particular arteries. Since the multi-compartment model represents position information roughly but in general does not signify the nonlinear convective acceleration term of 1D model, this model is usually seen as the first order discretization of the one-dimensional linear model [43].

### Applications

Mono-compartment models are simplified descriptions of an arterial system, simulating physiological properties of the arterial vessels with a few parameters. Multi-compartment models construct a full arterial network by connecting several mono-compartment models, describing particular information of different vessel compartments. Due to the simplicity of mono-compartment and multi-compartment models, merely a few researchers used them to reconstruct central aortic pressure.

In Windkessel models, proximal flow or peripheral pressure measurements were frequently used as inflow condition. Many researchers chose aortic flow as model input [22, 44, 45]. Flow at other positions was selected as model input, such as carotid flow [46], left ventricle flow [23] and mitral valve mean flow [47]. Few researchers chose brachial and radial pressure as model input [48]. For model parameter acquisition, the majority of researchers calculated model parameters by population averaging [22, 23, 44–46, 48] and only a small number of researchers adopted partially individualized model parameters [47].

Mono-compartment models are more commonly applied to the estimation of central aortic pressure than multi-compartment models. For example, the pressure waveform in the aorta was reconstructed by Stergiopulos et al. [44] and Struijk et al. [45] using a two-element Windkessel model. Cai et al. [22], Zala et al. [46] and Vennin et al. [22] employed a three-element Windkessel model to calculate central aortic pressure. A four-element Windkessel model was adopted by Her et al. [23] to reproduce the patient’s aortic pressure waveform undergoing counter-pulsation control by ventricular assist devices. Revie et al. [47] used a six-chamber lumped parameter model consisting of left ventricle and right ventricle, aorta, pulmonary artery, pulmonary vena cava and pulmonary vein to monitor aortic pressure changes. It was verified in clinical data [22, 23, 47] that Windkessel models can describe the general shape of pressure waveform in the ascending aorta, however, it is difficult to show the details of pressure waveform such as dicrotic notch features. Therefore, Windkessel models have limited accuracy for the estimation of central aortic pressure.

## 1D models

1D models are distributed parameter models. Theory and application of 1D models are described in this part, respectively. 1D models mainly focus on methods that solve one-dimensional equations and boundary conditions (including inflow, bifurcation and outflow conditions).

### Model descriptions

#### Model derivations

The one-dimensional arterial flow theory was proposed by some researchers [16, 17]. Euler [49] first established a 1D model using the one-dimensional theory. Although assumptions of the model are simple, it laid foundations for further studies. Reymond et al. [50] extended the existing 1D model to a more detailed model consisting of the foot and hand circulation. Moreover, the ventricular-arterial coupling model was developed and 1D model of the circulation was validated. 1D models are commonly used to represent pulse wave propagation phenomena of large arteries [29]. In the 1D model, the blood is assumed to be an incompressible Newton fluid and the vessel is an axisymmetric cylindrical tube. The 1D model is governed by two equations [51]. A continuity equation (see Eq. 1) and a momentum equation (see Eq. 2) both together describe the motion of the blood flow and the vessel wall. The formulas are as follows

1 $$\begin{aligned} \frac{\partial q}{\partial x}+\frac{\partial A}{\partial t}= & {} 0. \end{aligned}$$

2 $$\begin{aligned} \frac{\partial q}{\partial t}+\frac{4}{3}\frac{\partial \frac{q^2}{A}}{\partial x}= & {} -\frac{A}{\rho }\frac{\partial p}{\partial x}-\frac{8\mu }{\rho r^2}q. \end{aligned}$$

where *x* is the distance along the vessel, *t* is time, *q* is the blood flow rate, *p* is the blood pressure, *A* is the cross-sectional area, *r* is the vessel radius, $\rho$ is the blood density and $\mu$ is the viscosity.

#### Solving methods

For solving 1D Navier–Stokes equations, there are two types of methods including time domain and frequency domain methods. The time domain method can solve linear or nonlinear equations and frequency domain method can only solve linear equations.

##### a. Time domain method

Generally, Navier–Stokes equations of 1D models are nonlinear, which are solved in the time domain using numerical methods. At present, there are many numerical methods for solving the partial differential equations. Each method has its scope of application. The method of characteristics, finite difference method, finite volume method, finite element method and spectral method are frequently used to solve 1D pulse propagation equations.

The method of characteristics is a basic method of solving the partial differential equations. The essence of this method is the integral along the characteristic line of the partial differential equations to simplify the form of equations. The characteristic method has a clear physical meaning and a wide application scope. As for solving the differential equations of three independent variables, the method of characteristic can be very complicated, and there are still some problems to be solved. The governing equations can be solved by taking use of the method of characteristics [52–54].

The finite difference method is a numerical method for solving complex partial differential equations by approximating the derivatives with finite differences. While the principle of the finite difference method is simple, it can give the corresponding difference equation for any complex partial differential equation. Difference equations can only be considered as mathematical approximations of differential equations. This method has been used by a number of researchers. For instance, Olufsen et al. applied the two-step Lax–Wendroff method to solving the continuity and momentum equations [18, 19].

A finite volume method is developed on the basis of the finite difference method. To begin with, the calculated region is divided into a series of control volumes and there is a control volume at the surrounding of each grid point. Then each control volume is integrated and a set of discrete equations are obtained. Finally, the discrete equations need to be solved. The finite volume method is suitable for the computation of fluid. This method has a high computing speed and low requirements for the grid, but its precision is limited. To solve the differential equations, the finite volume method is often used [55, 56].

The finite element method uses the variational principle to minimize the error function. The advantage of the finite element method is that this method can simulate complex curve or surface boundary accurately. Furthermore, the division of the grid is arbitrary and it can design the general program easily. Nevertheless, the finite element method cannot give a reasonable physical explanation and some errors in the calculation are still difficult to improve. Recently, some investigators used the finite element method to solve the differential equations [57–59].

The spectral method is a class of computing techniques of using an orthogonal function or intrinsic function as an approximate function to solve certain differential equations. The superiorities of the spectral method are to obtain a higher precision using fewer grid points. The poor stability and high complexity in the treatment of boundary conditions are the major weaknesses of this method. The spectral method has been utilized to solve the one-dimensional pulse wave propagation equations by a few researchers [60, 61].

##### b. Frequency domain method

**Fig. 4:**
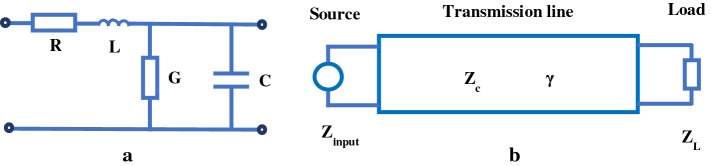
Transmission line equivalent circuit. **a** Arterial segment of unit length; **b** transmission line segment. Where $Z_{input}$ is the input impedance, $Z_L$ is the terminal impedance, $\gamma = \sqrt{(R+jwL)(G+jwC)}$ is the propagation constant and $Z_c= \sqrt{(R+jwL)/(G+jwC)}$ is the characteristic impedance. When the transmission line is lossless ($R=G=0$), $\gamma = w \sqrt{LC}$ and $Z_c= \sqrt{L/C}$

In order to reduce the computational complexity of the nonlinear model, a transmission line method is used to solve the Navier–Stokes equations in the frequency domain. The method requires that the 1D Navier–Stokes equations are linearized. According to the similarity of electromagnetic propagation theory and pulse wave propagation theory, linear 1D Navier–Stokes equations (see Eqs. 3, 4) in hemodynamics are converted into electrical transmission line equations (see Eqs. 5, 6) in the circuit [62]. The subsequent work is that we can employ methods of solving electric circuit to solve 1D Navier–Stokes equations. A transmission line equivalent circuit of an arterial segment is represented as shown in Fig. 4.

3 $$\begin{aligned} -\frac{\partial q}{\partial x}= \, {} \frac{dA}{dp}\frac{\partial p}{\partial t}=0. \end{aligned}$$

4 $$\begin{aligned} -\frac{\partial p}{\partial x}= \, {} \frac{\rho }{A}\frac{\partial q}{\partial t}+\frac{8\mu }{\pi r^2}q. \end{aligned}$$

5 $$\begin{aligned} -\frac{\partial I}{\partial x}= \, {} UG+C\frac{\partial U}{\partial t}. \end{aligned}$$

6 $$\begin{aligned} -\frac{\partial U}{\partial x}= \, {} IR+L\frac{\partial I}{\partial t}. \end{aligned}$$

where *U* is the voltage, *I* is the current, $R=\frac{8\mu }{\pi r^4}$ is the resistance, $L=\frac{\rho }{A}=\frac{\rho }{\pi r^2}$ is the inductance, $C=\frac{dA}{dp}=\frac{3 \pi r^2}{2Eh}$ is the capacitance, *E* is the Young’s modulus, and *h* is the arterial wall thickness. *G* is the conductance, describing blood flow leakage, which is usually neglected. The electrical circuit is comprised of resistive, inductive and capacitive elements. The values of these elements are calculated from mechanical and geometric parameters in the arterial tree.

#### Boundary conditions

For 1D pulse wave propagation equations, boundary conditions, commonly including inflow, bifurcation and outflow boundary conditions, need to be determined.

##### a. Inflow conditions

The flow waveform measured in vivo at ascending aorta or aortic root serves as the inflow condition using a magnetic resonance imaging or ultrasound equipment. Alternatively, a flow function derived from a simple model of the heart can serve as the inflow condition. The function is periodic which is mainly determined by the cardiac period and the cardiac output parameters [18].

7 $$\begin{aligned} q(0,t)= \, {} CO\frac{t}{\tau ^2}e^{\frac{-t^2}{2\tau ^2}}, \quad 0\le t<T. \end{aligned}$$

8 $$\begin{aligned} q(0,t+jT)= \, {} q(0,t), \quad j=1,2,3,\ldots \end{aligned}$$

where *CO* denotes the cardiac output, $\tau$ denotes the time for cardiac output to reach its maximum and *T* denotes the cardiac period.

The other inflow condition is that the left ventricle of the heart is coupled to 1D arterial tree model. Two main heart models are developed to represent the relationship between the ventricular pressure and volume. The time-varying elastance model is that the heart is seen as an elastance varying with time [63]. This left ventricular model indicates the instantaneous change of pressure and volume in left ventricle.

9 $$\begin{aligned} E(t)=\frac{P(t)}{V(t)-V_0}. \end{aligned}$$

where *E* is the elastance, *P* is the instantaneous pressure of left ventricle, *V* is the instantaneous volume of left ventricle and $V_0$ is the volume intercept of the end-systolic line.

The one-fiber model is another heart model in which the heart is described as a rotationally-symmetric cylindrical or spherical cavity [64]. The left-ventricular pump function is reflected by wall tissue function. It is assumed that fiber stress and strain are homogeneously distributed in the thick wall. The ratio of the fiber stress ($\tau _f$) to the pressure of the left ventricle ($P_{lv}$) is closely related to the ratio of the wall volume ($V_w$) and the cavity volume ($V_{lv}$).

10 $$\begin{aligned} \frac{P_{lv}}{\tau _f}=\frac{1}{3}ln\left( 1+\frac{V_w}{V_{lv}}\right) . \end{aligned}$$

##### b. Bifurcation conditions

In arterial networks, vessel branching is another important sort of boundary condition. At the bifurcation, the principle of pressure and flow continuity was applied [32, 55, 59, 65]. It is assumed that all bifurcations are situated at a point and the effect of the bifurcation angles is ignored. Without any blood leakage, according to the conservation of mass, the outlet flow of a parent vessel is equal to the sum of the inlet flow of two daughter vessels at the bifurcation (see Eq. 11). Considering the continuity of pressure, the pressure of a parent vessel and the pressure of each daughter vessel are identical at the bifurcation (see Eq. 12). In reality, there exist energy losses at the bifurcations. Generally, the energy loss at a bifurcation is quite small and they are often neglected. For the bifurcation at the aortic arch, however, large energy losses are brought about. Because it has a nearly right-angled turn and a high velocity of blood at this bifurcation, remarkable vortices are produced. In order to represent the loss at the aortic arch bifurcation, a loss coefficient *K* is introduced [19] (see Eq. 13).

11 $$\begin{aligned} q_{pa}= \, {} q_{d1}+q_{d2}. \end{aligned}$$

12 $$\begin{aligned} p_{pa}= \, {} p_{d1}=p_{d2}. \end{aligned}$$

13 $$\begin{aligned} p_{di}= \, {} p_{pa}+\frac{\rho }{2}((\bar{u_x})^2_{pa}- (\bar{u_x})^2_{di})-\frac{K_{di}}{2}(\bar{u_x})^2_{pa}, \quad i=1,2. \end{aligned}$$

where $\bar{u_x}$ denotes the average axial velocity and $\rho$ denotes the density. The subscripts *pa* and *d* indicate the parent and the daughter vessel, respectively.

##### c. Outflow conditions

The simplest outflow boundary condition is that the terminal of vessel is seen as a pure resistive load [18]. Nevertheless, a precise peripheral resistance value is not easy to be determined. Assuming a constant relation between pressure and flow, pressure and flow are in phase, actually, which is not physiologically reasonable for large arteries. The pure resistance model is merely suitable for small arteries. To overcome the weaknesses, a phase-shift between pressure and flow should be applied to the downstream boundary. The terminal impedance for the pure resistance is as follows.

14 $$\begin{aligned} Z_L(w)=R_T \end{aligned}$$

where $Z_L(w)$ denotes the terminal impedance of large arteries and $R_T$ denotes the peripheral resistance.

The distal network of truncated vessel is represented as the terminal impedance which is modeled by a three-element Windkessel model [66]. The three-element Windkessel model is made up of a resistance $R_1$ in series with a parallel combination of a capacitor $C_T$ and another resistance $R_2$. This model cannot represent the wave propagation effects. The frequency dependent impedance of Windkessel model is given by

15 $$\begin{aligned} Z_L(w)=\frac{R_1+R_2+iwC_TR_1R_2}{1+iwC_TR_2}. \end{aligned}$$

The relation between pressure and flow at the truncated arteries is given by the following differential equation.

16 $$\begin{aligned} \frac{\partial p}{\partial t}=R_1\frac{\partial q}{\partial t}-\frac{p}{R_2C_T}+\frac{q(R_1+R_2)}{R_2C_T}. \end{aligned}$$

In recent years, the structured-tree model presented by Olufsen [67] has become a popular outflow boundary condition. Compared with the resistance and Windkessel model, the structured-tree model can simulate the impedances of small arteries more accurately. At the terminal branches of the truncated arterial tree, a structured-tree model, which is based on linear one-dimensional Navier–Stokes equations, provides a dynamic boundary condition for large arteries. The model can describe the phase lag between pressure and flow and the high frequency oscillations. Meanwhile, it can also represent the wave propagation effects of arterial system. According to the convolution theorem, the outflo w boundary condition is obtained by

17 $$\begin{aligned} p(x,t)=\frac{1}{T}\int _{-T/2}^{T/2}z(x,t-\tau )q(x,\tau )d\tau . \end{aligned}$$

The root impedance is computed from the relationship between pressure and flow as follow

18 $$\begin{aligned} Z_L(w)=\frac{ig^{-1}sin(wL/c)+Z(L,w)cos(wL/c)}{cos(wL/c)+igZ(L,w)sin(wL/c)}. \end{aligned}$$

where $g=cC$, *c* denotes the wave propagation velocity, $Z_L(w)$ denotes the root impedance, namely, the terminal impedance of large arteries, *Z*(*L*, *w*) denotes the terminal impedance of small arteries, *L* denotes the vessel length and *w* denotes the angular frequency.

### Applications

1D models can simulate pressure and flow waveforms at any point of the arterial network according to their distributed properties. Since 1D models include too many vascular parameters, they haven’t been extensively used to reconstruct central aortic pressure up to now. By limiting the number of personalized parameters, 1D models may have a great potential for estimation of central aortic pressure in clinical practice.

For 1D models, aortic flow waveform is the most common inflow condition [24, 68]. Few researchers used peripheral pressure measurement (e.g. brachial pressure or radial pressure) as model input [25, 69]. The aortic flow waveform can be measured by ultrasound equipment, however, it is not accurate. Meanwhile, it is very expensive to obtain aortic flow waveforms by MRI equipment. The pressure waveforms of good stability can readily be recorded using peripheral pressure sensors such as applanation tonometry. Generally, vascular geometric parameters of 1D models are measured by MRI or CT equipment, which is costly and complex. Many researchers used population averages for these geometric parameters. Nevertheless, Harana et al. [24] measured aortic geometry parameters including ascending aorta, descending aorta and three supra-aortic branches using MRI equipment. Meanwhile, pulse wave velocity (PWV) and vascular resistance and compliance parameters for each subject were calculated from measured data. Remaining blood flow parameters such as density and viscosity were assumed to be constants.

In recent years, 1D models with different degrees of complexity have been utilized by several researchers to reconstruct central aortic pressure. For example, Bárdossy et al. [69] presented a “backward” calculation method to derive central aortic pressure waveform in a 1D model comprising 50-segment arteries. A personalized transfer function between aorta and radial was established by Jiang et al. [24] to estimate central aortic pressure based on 1D model including 55 large arteries and 28 small arteries. Khalifé et al. [68] estimated absolute pressure in the aorta by combining a reduced 1D model including an ascending aorta branch and a descending aorta branch with MRI. A non-invasive personalized estimation method of central aortic pressure was developed by Harana et al. [24] using a 1D aortic blood flow model. Owing to the complexity of 1D models, the details of pressure waveform can be described easily [24]. If all vascular geometric parameters are measured by noninvasive equipments, 1D models can provide accurate estimation of central aortic pressure.

## Tube-load models

Tube-load models are distributed parameter models. Theory and application of tube-load models are described in this part, respectively. Tube-load models mainly focus on various tube models based on different assumptions.

### Model descriptions

**Fig. 5:**
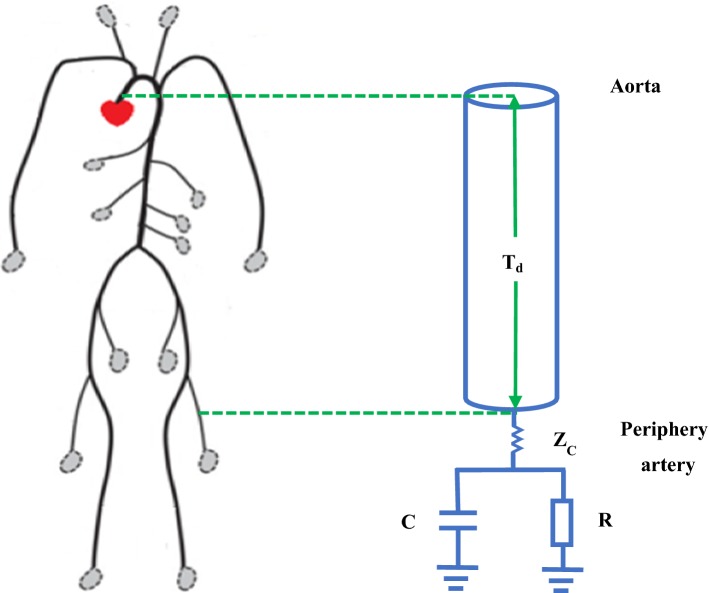
Single tube model with a load. *R* the peripheral resistance, *C* the load compliance, $Z_c$ the characteristic impedance and $T_d$ the time delay

Tube-load models are a kind of highly simplified transmission line models, which are made up of multiple parallel tubes with loads [70]. The simplest tube-load model whose tube is taken as lossless, linear and uniform, only consists of a tube and a load as shown in Fig. 5. The tube signifies the wave transmission pathway of large arteries and the load signifies the wave refection site in the arterial terminal. The formulas are as follows.

19 $$\begin{aligned} T_d = \, {} \sqrt{LC} \end{aligned}$$

20 $$\begin{aligned} Z_c = \, {} \sqrt{L/C} \end{aligned}$$

21 $$\begin{aligned} \Gamma (jw) = \, {} \frac{Z_L(jw)-Z_c}{Z_L(jw)+Z_c} \end{aligned}$$

22 $$\begin{aligned} P(x,jw) = \, {} P_f(0,jw)e^{jwT_d x/d}+P_b(0,jw)e^{-jwT_d x/d} \end{aligned}$$

23 $$\begin{aligned} Q(x,jw) = \, {} \frac{1}{Z_c}(P_f(0,jw)e^{jwT_d x/d}+P_b(0,jw)e^{-jwT_d x/d}) \end{aligned}$$

24 $$\begin{aligned} P_p(jw) = \, {} \frac{\left( jw+\frac{1}{RC}+\frac{1}{2Z_c C}\right) e^{jwT_d} +\frac{1}{2Z_c C}e^{-jwT_d}}{jw+\frac{1}{RC}+\frac{1}{Z_c C}}P_c(jw) \end{aligned}$$

where *L* denotes the large artery inertance, *C* denotes the large artery compliance, *d* denotes length of tube, $T_d$ denotes the time delay, $Z_c$ denotes the characteristic impedance, $Z_L$ denotes the terminal impedance, $\Gamma$ denotes the wave reflection coefficient, *P* denotes the pressure, *Q* denotes the flow. In addition, the subscripts *f*, *b*, *c* and *p* are for the forward and backward wave, aorta and peripheral artery, respectively.

For tube-load models, there are three main types of loads, including pure resistance, generic pole-zero, three-element Windkessel models. The pure resistance load is a high simplification of small arterial vessels, accounting for the peripheral resistance [26, 71]. The greatest advantage of this kind of load is simplicity and its disadvantage is that there is a big difference from the real vascular structure. The generic pole-zero models as a terminal impedance can change the order of system flexibly [72]. However, the weakness of the model is that model parameters do not have physiological significance. The most frequently used load is the three-element Windkessel model, which consists of a characteristic impedance, a resistance and a compliance [73, 74]. Although the Windkessel model fails to provide the detailed anatomical and mechanical information of arterial network, it can describe the lumped properties of terminal arterial vessels well.

Based on different assumptions, tube-load models have been developed into T-tube, lossy tube-load, nonlinear tube-load and non-uniform tube-load models, which are summarized in Table 3. In comparison with the simplest tube-load model, these models have a higher accuracy in hemodynamic simulation.

**Table 3:** Summary of tube-load models based on different assumptions

Model type	Model feature	Model parameter estimation
T-tube model	Two parallel tubes signify head-end and body-end propagation paths	Using frequency domain method from central and peripheral pulse waves
Lossy tube-load model	Blood pressure decays along the arterial tree	Using frequency domain method from central and peripheral pulse waves
Nonlinear tube-load model	Arterial compliance is an exponential function of blood pressure	Using time domain method from central and peripheral pulse waves
Non-uniform tube-load model	Tube exponentially tapers along arterial vessels	Using frequency domain method from central and peripheral pulse waves

#### T-tube model

**Fig. 6:**
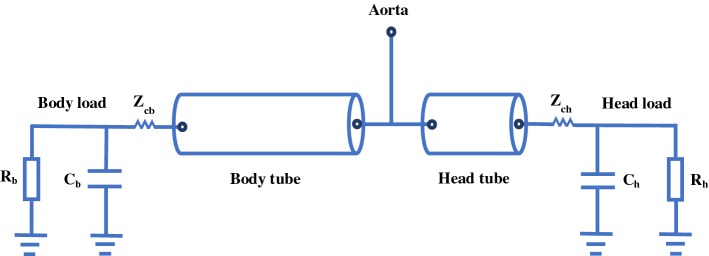
The T-tube model. *R* the peripheral resistance, *C* the load compliance, $Z_c$ the characteristic impedance; subscripts b and h are for the body load and head load, respectively

The T-tube model is a frequently-used tube-load model, in which there are two tubes with two terminal loads as shown in Fig. 6 [75, 76]. While the tubes signify head-end and body-end travel paths, the loads represent head-end and body-end reflection sites, respectively. The advantages of the T-tube model are that it is a simple model and that it can indicate main features of pressure and flow waveforms in the large blood vessels. However, it cannot represent the wave reflection intensely depending on frequency.

#### Lossy tube-load model

**Fig. 7:**
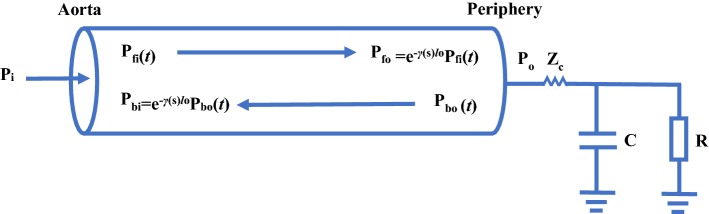
The lossy tube-load model. *P* the blood pressure, $\gamma$ the wave propagation constant, $l_0$ the length of the tube, *R* the peripheral resistance, *C* the load compliance, $Z_c$ the characteristic impedance; subscripts i and o are for the inlet and outlet of the tube, respectively

Tube-load models mentioned above are based on lossless tube-load models. The lossless tube-load model is a kind of ideal and simple model. In some situations, ignoring the loss of tube can bring large errors [77–80]. For example, for reconstructing the central aortic pressure waveform from peripheral pressure waveforms, it is generally assumed that the mean pressure is same at any position. In fact, the blood pressure loss is large along the arterial tree in pathophysiologic conditions or postoperative period. In order to improve the accuracy of tube-load models, Abdollahzade et al. [20] proposed the lossy tube-load model of arterial tree in humans as shown in Fig. 7. Compared with lossless tube-load models, lossy tube-load models have smaller errors and larger efficacy.

#### Nonlinear tube-load model

**Fig. 8:**
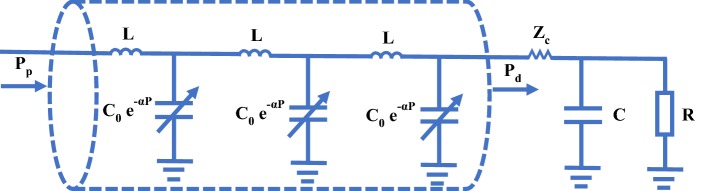
The nonlinear tube-load model. *P* the blood pressure, $\alpha$ the constant, *L* the large artery inertance, *R* the peripheral resistance, $C_0$ the large artery compliance, *C* the load compliance, $Z_c$ the characteristic impedance; subscripts p and d are for the inlet and outlet of the tube, respectively

Previously, tube-load models were regarded as linear models. In recent years, Gao et al. [81, 82] has developed a nonlinear tube-load model based on the exponential relationship between blood pressure and compliance as shown in Fig. 8. In the nonlinear tube-load model, arterial compliance is no longer a constant but a function of blood pressure. In contrast with the linear tube-load model, the nonlinear tube-load model has a higher accuracy in estimating pulse transit time [21].

#### Non-uniform tube-load model

**Fig. 9:**
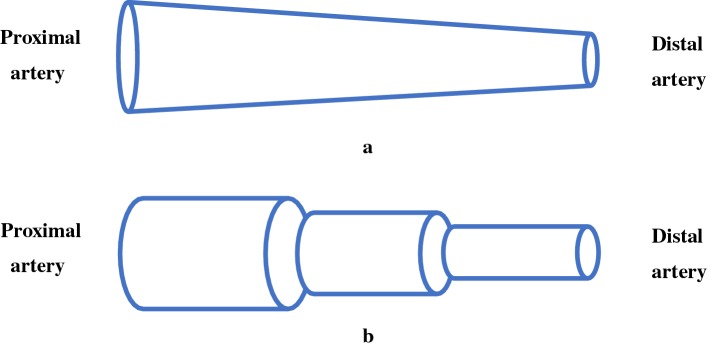
The non-uniform tube-load model. **a** A non-uniform tube tapering with the position exponentially; **b** a non-uniform tube consisting of multiple uniform tubes with a successive decrease in diameter dimension

The electrical transmission line theory is applied to mathematical modeling of arterial vessels. It is generally assumed that the transmission line model is a uniform tube with a terminal load. Taylor [83] explored the wave propagation properties of a non-uniform transmission line, since the uniform tube is too simple to reflect the real characteristics of the arteries. Subsequently, Einav et al. [84] proposed an exponentially tapered transmission line model of the arterial system, in which the geometrical properties and wall elasticity of the tube exponentially tapered along the length of arterial vessels. At present, there are two methods to describe the taper effects of a non-uniform tube. One method is that the inductance and capacitance of the tube change with the position exponentially as shown in Fig. 9a [85–87]. Another method is that an artery is separated into several smaller segments and each segment is viewed as a uniform tube as shown in Fig. 9b [88, 89].

### Applications

Tube-load models include tubes and loads, describing wave propagation and refection phenomenon with only a few parameters. Combining the advantages of Windkessel models (simplicity) and 1D models (accuracy), tube-load models have become an attractive tool for the reconstruction of central aortic pressure waveform.

For tube-load models, inflow conditions are obtained from one or two measured peripheral pressure waveforms. A radial [71, 73, 74], brachial [26] or femoral [72] pressure measurement is commonly used as model input. Moreover, some researchers [27, 90–92] chose two measured peripheral pressure waveforms as inflow conditions such as radial and femoral arteries. Because intravascular and extravascular pressure at radial artery is very close, the radial pressure waveform can be accurately recorded using an applanation tonometry. The brachial cuff-based measurement is a very convenient approach for obtaining brachial pressure, especially suitable for longer term outpatient monitoring of central aortic pressure or potential general hospital ward usage.

Single tube or T-tube models with different loads are used to described the relation between central and peripheral pressures. For example, Swamy et al. [72] employed a single tube with a generic pole-zero load to establish an adaptive transfer function between aortic and femoral pressures. A single tube model with a resistance load was applied by Gao et al. [71] and Natarajan et al. [26] to the estimation of central aortic pressure. Individualized transfer functions were built by Sugimachi et al. [73] and Hahn et al. [74] using a single tube model with a three element Windkessel load. Ghasemi et al. [27, 90], Lee [91] and Kim et al. [92] utilized a T-tube model with a three-element Windkessel load, to reconstruct central aortic pressure from two measured peripheral pressure.

In order to acquire an adaptive or individualized transfer function, most researchers measured the pulse transit time for each subject through some noninvasive approaches and calculated the remaining parameters by population averaging [26, 27, 71–74, 92, 93]. Blind system identification is another method of estimating model parameters, which can reconstruct the central aortic pressure waveform from two distinct peripheral pressure waveforms [90, 91]. The blind system identification method can obtain fully individualized parameters. The weakness of this method is that it is inconvenient to measure multiple distinct peripheral pressure waveforms in clinical practice. To examine the performance of individualization, Hahn [93] made a comparative study on the estimation of central pressure among a fully individualized, two partially individualized and a fully generalized transfer functions based on tube-load models. The 9 swine experiment results showed that the fully individualized transfer function had higher accuracy than two partially individualized functions and the fully nonindividualized transfer function. Since in the tube-load model, only one parameter, the pulse transit time, could be readily individualized, tube-load models had moderate accuracy for estimation of central aortic pressure.

## Discussions and conclusions

### Comparisons of three types of models

In this paper, recent progresses of Windkessel, 1D and tube-load models in the arterial system are reviewed. Windkessel models are developed into increasingly complicated and detailed structures and a variety of Windkessel models are established [39, 94]. 1D models including more arterial segments and coupling the heart have been set up in recent years [29, 50]. Tube-load models with various types of tubes based on different assumptions are investigated [77, 81, 83].

To select an appropriate model, the comparisons among three types of low-dimensional models are made. The Windkessel model can give a global description of the arterial system and every model element has a particular physiological meaning [15, 28]. Windkessel models only include a few parameters and the parameters are usually estimated from measured aortic pressure and flow waveforms. Due to its simplicity, the Windkessel models have a low accuracy. The iterative and system identification techniques are adopted by most researchers, such as linear least-squares method [95] and subspace model identification method [96]. Since the Windkessel model is established by a set of ordinary differential equations, this model is simpler than the distributed parameter model built by a set of partial differential equations. The model is fit for calculating hemodynamic parameters and simulating the whole circulatory system.

1D models and tube-load models are distributed parameter models which signify the distributed properties along the arterial vessels. 1D models can accurately predict flow and pressure in the entire arterial tree and the model parameters can truly reflect the physiological properties of arterial vessels [49]. A 1D model is determined by a set of partial differential equations with a lot of parameters. It is very difficult to determine a large number of parameters using system identification methods. The majority of geometrical and mechanical parameters can be directly measured by MRI, CT or Doppler ultrasound equipment. The rest of the parameters are approximated or seen as constants such as the thickness of arterial wall and width of boundary layer. The 1D model is an appropriate approach to study pulse wave propagation phenomenon in arterial system.

Tube-load models are a parallel connection of multiple tubes with parametric loads, which is a simplified 1D model. The tube indicates the path of pulse wave propagation and the load indicates the effective reflection point [75, 76]. The transit time of pressure and flow waves is described by the time delay constant parameter of the model. Tube-load models can represent the relationship between central and peripheral arteries with a few parameters. Once proximal and distal waveforms of the arterial tree are obtained, the parameters of tube-load models can be determined by system identification techniques. In comparison with 1D model, tube-load model has a lower computation cost. Meanwhile, the shortage of the tube-load model is that it is less accurate than 1D model. Tube-load models are suitable for investigating wave propagation and reflection.

In general, computational time of three types of models is short and they have the potential for real-time and general use clinical monitoring in intensive care. In specific applications, however, in order to obtain more accurate clinical parameters, these models may need to be optimized such as personalization of the model which will be made a detailed discussion later. The personalized model requires that model parameters can be obtained individually. The real-time character depends on required patient-specific model parameters, which should be discussed in the following two cases. If the patient-specific model parameters cannot be real-time obtained, the central aortic pressure monitoring is impossible. For example, the pulse transit time parameter in tube-load models can be obtained from various combinations of physiological signals such as two pulse waveforms (e.g. carotid and radial sites) and a combination of ECG and pulse waveforms (e.g. radial site) methods. If we choose the former method (two pulse waveforms), the pulse transit time parameter cannot be real-time obtained since the carotid pulse waveform is difficult to measure for long time. In this case, we cannot monitor central aortic pressure in real-time. The pulse transit time parameter can be real-time estimated if we choose the latter method (ECG and pulse waveforms) because ECG and radial pulse waveforms can be measured simultaneously for long time, real-time central aortic pressure can be monitored. Windkessel models and tube-load models can be used in long-term monitoring of central aortic pressure/potential general hospital ward usage, however, 1D models cannot. This is because geometric parameters of 1D model need to be measured by CT or MRI equipment, which is very complex and costly. Detailed comparisons of three types of low-dimensional models are summarized in Table 4.

**Table 4:** Comparison of Windkessel, 1D and tube-load models

Model type	Windkessel models	1D models	Tube-load models
Model structure	Including capacitors, resistors and inductance	Dividing the arterial tree into many small segments	Consisting of multiple tubes with terminal loads
Model parameters	Few	A lot	Few
	Easy to estimate	Difficult to estimate	Easy to estimate
Complexity	Low	High	Moderate
Accuracy	Low	High	Moderate
Simulate wave propagation and reflection phenomenon	No	Yes	Yes
Inflow condition	Aortic flow function	Aortic flow or pressure waveforms	Aortic pressure waveforms
	Aortic pressure waveforms	Aortic flow function	
		Heart model	
Outflow condition	Venous pressure	Pure resistance	Windkessel model
		Windkessel model	Generic pole-zero model
		Structured-tree model	Pressure
		Pressure	
Geometrical and mechanical properties	None	Vessel diameter, length, thickness, elasticity and blood viscosity	Vessel length and elasticity

### Future challenges

Although a variety of physics-based models have been developed, there still exist a number of challenging problems to be solved. Multi-scale modeling, coupling of various systems and patient-specific modeling are very significant research subjects at present.

#### Multi-scale modeling

A model of each scale has its scope of application [29, 97, 98]. Low-dimensional models have low computational cost but poor accuracy, which is applicable to represent the global properties of the arterial networks. Nevertheless, high-dimensional models can offer high accuracy simulation but with greater complexity. They are commonly used to describe the local properties of arterial vessels in detail. Therefore, coupling models of various different scales can combine the advantages of different dimensional models [43, 99, 100]. Multi-scale modeling of arterial vessels can be a powerful tool for providing potential applications in clinical practice.

#### Coupling of various systems

There exist a variety of biological systems such as the nervous system and the respiratory system in human body which run simultaneously and interactively. The effects of other biological systems are usually ignored in physical modeling of cardiovascular system. As a matter of fact, the nervous system has a significant impact on cardiovascular system [101]. For example, as the blood pressure changes, to avoid dysfunctions, the sympathetic nerves and the parasympathetic nerves are usually motivated to regulate cardiovascular response [101]. Smith et al. [102] proposed a modified cardiovascular model by including a minimal autonomic nervous system activation model. This modified model can simulate various cardiovascular diseases such as hypovolemic shock septic shock, cardiogenic shock, pericardial tamponade and pulmonary embolism.

Due to the fact that both the respiratory system and the cardiovascular system located in the thoracic cavity, the cardiovascular system can be influenced by the respiratory system strongly [103]. For example, an integrated model of the cardiopulmonary system was present by Albanese et al. [104], which included cardiovascular circulation, respiratory mechanics, gas exchange and neural control mechanisms. The physiological parameters in normal and pathological conditions were simulated and the interactions between the cardiovascular and respiratory systems were explained. Trenhago et al. [105] proposed a refined coupled model of the cardiovascular and respiratory systems, consisting of 19 compartments, in which the respiratory system was extended to include a complex system for gas exchange and transport. The advantage of the refined model is that it enables to simulate situations in which existing models cannot predict mimic and it helps us to understand complex mechanisms better. For modeling of cardiovascular system, the respiratory effects should be considered. In comparison with previous cardiovascular model, a modified cardiovascular model integrating neurological and respiratory components has better accuracy. Hence, combining the nervous system and the respiratory system with the cardiovascular system might be a good way to improve the accuracy of models.

#### Patient-specific modeling

Patient-specific models can provide opportunities for improving accuracy [106–108]. The patient-specific parameters can be obtained from imaging techniques such as magnetic resonance imaging, computed tomography and ultrasound. A properly personalized model can predict physiological or pathological status more accurately [109, 110]. The personalized modeling in the arterial system can play an increasingly key role in the development of medical instruments.

This paper takes the estimation of central arterial pressure as an example to introduce personalization of physics-based models. Although several individualized estimation methods of central aortic pressure have been proposed, these methods haven’t been sufficiently verified in clinical practice yet. An accurate and convenient method of reconstructing central aortic pressure waveform with sufficient verification is a current hot topic. The physics-based models with clear physiological meaning may provide great potential for individualized estimation approach of central aortic pressure. In these three types of physics-based models, 1D models are the most accurate and complicated. Under the condition of guaranteeing a high accuracy, reducing the complexity of the model as much as possible is the prefered method. By examining the influence of the complexity of the arterial tree on the accuracy of the model, the complex model can be greatly simplified. For example, a 1D aortic model consisting of ascending aorta, aortic arch, thoracic aorta and abdominal aorta with Windkessel outflow conditions may be created to reconstruct aortic pressure waveform with a high accuracy. Another feasible way is to use body size parameters to replace complex blood vessel parameters. Since 1D models have too many parameters, it is actually impossible to measure all parameters, however, human body size parameters are available easily. A statistical analysis has shown that the blood vessel sizes have close correlation with sex, age, height, and weight of a subject [111]. Furthermore, Young’s modulus representing vascular stiffness has a strong correlation with age. It might be possible to build relationships between geometrical and mechanical parameters of 1D models and body size parameters of the subject. The geometrical and mechanical parameters can be firstly employed by population averages and then the average parameters can be calibrated with body size parameters. Combining with the two methods above, a modified 1D model may be the best choice for estimating central aortic pressure.

### Conclusion

In conclusion, different physics-based models in cardiovascular system have different traits and the selection of models mainly depends on the aim of modeling including complexity and accuracy required. By discussing the advantages and disadvantages of various physics-based models, this review contributes to a better understanding of physiological mechanism in the arterial system and provides effective guidance on low-dimensional physics-based modeling.
